# MRSA admission burden and acquision in a tertiary care hospital

**DOI:** 10.1186/1753-6561-5-S6-O80

**Published:** 2011-06-29

**Authors:** H-MA Schmidt, E Ryan, MW Maley, SJ van Hal

**Affiliations:** 1 Microbiology and Infectious Diseases, Sydney South West Pathology Service, Liverpool Hospital, Sydney, Australia; 2 Infection Prevention Unit, Liverpool Hospital, Sydney, Australia

## Introduction / objectives

The burden of methicillin-resistant *Staphylococcus aureus* (MRSA) impacts on the effectiveness of infection control strategies to prevent MRSA transmission. Few studies examining the admission and acquisition burden of MRSA exist. We investigated the prevalence of MRSA in patients admitted to a tertiary referral hospital that does not conduct universal screening, to determine the MRSA admission burden and acquisition rate.

## Methods

Nasal swabs were collected for MRSA culture on admission (n=1159) and discharge (n=373) from patients admitted to acute adult wards. Strain typing for similarity was performed using antibiograms and pulsed field gel electrophoresis (PFGE).

## Results

The overall MRSA admission burden was 10.9/100 admissions. This consisted of patients identified by nasal swabs (3.5/100 admissions) or clinical samples (1.1/100 admissions) collected within 48 hours of admission; and those with a known history of MRSA colonisation (6.2/100 admissions). During the study period, 1.7 new MRSA acquisitions per 100 admissions were detected in patients who had exit sampling. Of these, 83% represented colonisation events with the remaining 17% clinical infections. MRSA acquisition was associated with longer hospital stays (p<0.01).

## Conclusion

Despite a high MRSA admission burden, the rate of acquisition was modest. 67% of the total MRSA burden was identified without universal screening and could be isolated. However, existing strategies for controlling MRSA are resource and cost intensive. Furthermore, nasal carriage represents an unrecognised reservoir for the transmission of MRSA to new patients, the relative importance of which must be considered. Further exploration of the factors underlying the acquisition rate will assist in identifying strategies to prevent MRSA transmission.

## Disclosure of interest

None declared.

